# Clinical Implications of the Change in Glomerular Filtration Rate with Adrenergic Blockers in Patients with Morning Hypertension: The Japan Morning Surge-1 Study

**DOI:** 10.1155/2013/413469

**Published:** 2013-12-01

**Authors:** Seiichi Shibasaki, Kazuo Eguchi, Yoshio Matsui, Kazuyuki Shimada, Kazuomi Kario

**Affiliations:** Division of Cardiovascular Medicine, Department of Medicine, Jichi Medical University School of Medicine, 3311-1 Yakushiji, Shimotsuke, Tochigi 329-0498, Japan

## Abstract

*Background*. The aim of this study was to clarify the relationship between the change in estimated glomerular filtration rate (eGFR) and urinary albumin by antihypertensive treatment. *Methods*. We randomized 611 treated patients with morning hypertension into either an added treatment group, for whom doxazosin was added to the current medication, or a control group, who continued their current medications. We compared the change in eGFR and urinary albumin creatinine ratio (UACR) between the groups. *Results*. The extent of the reduction in eGFR was significantly greater in the added treatment group than in the control group (−3.83  versus −1.08 mL/min/1.73 m^2^, *P* = 0.001). In multivariable analyses, the change in eGFR was positively associated with the change in UACR in the added treatment group (*β* = 0.20, *P* = 0.001), but not in the control group (*β* = −0.002, *P* = 0.97). When the changes in eGFR were divided by each CKD stage, eGFR was significantly more decreased in stage 1 than in the other stages in the added treatment group (*P* < 0.001), but no differences were seen in the control group (*P* = 0.44). *Conclusion*. The reduction of eGFR could be seen only in the early stage of CKD, and this treatment appeared to have no negative effect on renal function.

## 1. Introduction

Kidney disease is increasing rapidly along with the increasing prevalence of hypertension and the aging of many societies worldwide [1]. A decline in the glomerular filtration rate (GFR) and an increase in albuminuria, established markers of kidney function, have been reported to be risk factors for various diseases, such as cardiovascular disease (CVD) [2–7], heart failure [6], cerebrovascular disease [4–6], hospitalization [7, 8], death from cardiovascular causes [9], and all-cause mortality [5–9]. Accordingly, the evaluation of GFR is of increasing importance in decisions about appropriate therapeutic strategies.

Many studies have shown that deterioration of GFR was attributable to hypertension [10–13]—in particular, a low GFR has frequently been observed in morning hypertension [13–15]. In addition, several studies have suggested that morning hypertension was caused by sympathetic nerve hyperactivity [16, 17]. Consequently, antihypertensive treatment which suppresses sympathetic nerve hyperactivity might be pathophysiologically effective for preserving renal function in patients with morning hypertension, as was proven in the case of renal sympathetic nerve ablation [18].

Aggressive blood pressure (BP) control has been recommended in hypertensive patients with CKD [19]. It was reported that GFR could be preserved in association with the reduction of urinary microalbumin by antihypertensive treatment [20], even with doxazosin [21]. However, several papers have demonstrated that urinary microalbumin was reduced despite the decrease in GFR in response to antihypertensive treatments [22, 23]. It has not been clarified how an aggressive antihypertensive treatment alters the GFR and urinary microalbumin and how these two parameters are related to each other during antihypertensive treatment. Furthermore, the effects of alpha or beta adrenergic blockers on these parameters are not well understood. Our hypotheses in this study were that the GFR would increase if morning hypertension was controlled by adrenergic blockers and that the change in GFR by adrenergic blockers would be associated with the improvement of urinary microalbumin. We performed this study to examine these hypotheses.

## 2. Methods

This study is a subanalysis of the Japan Morning Surge-1 (JMS-1) Study, which was conducted from August 2003 to August 2005 by 20 doctors at 16 institutions (7 primary practices, 8 hospital-based outpatient clinics, and 1 specialized university hospital) in Japan. This study was registered on 2 clinical trial registration sites: ClinicalTrials.gov: no. NCT00285519 and UMIN (University Hospital Medical Information Network) Clinical Trial Registry (UMIN-CTR): no. C000000309. The ethics committee of the internal review board of the Jichi Medical University School of Medicine, Japan, approved the protocol.

### 2.1. Study Design

The study protocol and design were described in our previous publication [24]. Briefly, patients who had been treated with various antihypertensive agents except for alpha or beta blockers were enrolled [25]. All patients were randomized either to a group in which morning BP was strictly moderated by a bedtime administration of doxazosin (the “added treatment group”) in addition to the patients' current hypertensive treatment regimen or to a control group whose members received no additional medication (the control group). There were no significant differences in antihypertensive agents between the groups. The randomization was carried out by an independent research center (Biomedis International, Ltd., Tokyo, Japan) by telephone. In the added treatment group, doxazosin was added just after randomization at a dose of 1 mg and titrated up to 4 mg (the recommended dose in Japan) over 3 months to achieve a morning systolic blood pressure (SBP) of less than 135 mmHg. If the morning SBP remained more than 135 mmHg at 3 months after starting doxazosin, a *β*-blocker (usually atenolol 25 mg/day) was added at bedtime. Patients in the control group continued their current medications throughout the study period even when they presented with high morning BP.

### 2.2. Patients

We enrolled 611 hypertensive patients whose morning SBP levels (the average of 6 readings: 2 measurements taken on each of 3 days) measured by home BP monitoring were more than 135 mmHg while being on stable antihypertensive medication for at least 3 months. They had never taken any *α*-blockers or *β*-blockers before this study. We excluded patients who had arrhythmias, a history of heart failure, orthostatic hypotension, dementia, malignancy, or chronic inflammatory disease. Written informed consent was obtained from all participants.

### 2.3. Blood Pressure Measurements

Morning BP was measured within 1 hour after waking, and before breakfast and taking antihypertensive medication, and evening BP was measured before taking antihypertensive medication and just before going to bed. A validated oscillometric device, the HEM-705IT (Omron Healthcare Inc., Kyoto, Japan) [26], was used based on the Japanese Hypertension Guidelines [27]. BP measurements were made on each occasion in the sitting position after a 30s interval. We asked study patients to write down each home BP value, and BP analysis was conducted using the average of 2 measurements taken over each of the 3 days (6 readings in total) in the sitting position. Office BP was measured using the same monitor (HEM-705IT), and the average of 2 consecutive measurements was used for analyses.

### 2.4. Blood and Urine Examinations

Blood and urine samples were collected in the morning in a fasting state at enrollment and at the end of the study (6 months). The urinary albumin excretion level was measured using the immunoturbidimetric method (Mitsubishi Chemical Iatron Inc., Tokyo, Japan) and expressed as the urinary albumin to creatinine ratio (UACR, mg/gCr). Both serum creatinine (s-Cr) and urine creatinine were measured by the Jaffe reaction without deproteinization and then quantified by a photometric method. The intra-/intercoefficients of variation were 1.52% and 2.48%, respectively, for the urinary albumin assay. The estimated GFR (eGFR) was calculated using a validated equation based on the Modification of Diet in Renal Disease (MDRD) study with a Japanese coefficient of 0.881: eGFR (mL/min/1.73 m^2^) = 0.881 × 186.3 × Age^−0.203^ × S-Cr^−1.154^ (if female × 0.742) [28]. The patients were classified into stages 1 to 5 based on the levels of GFR along with the lines of CKD staging [29]: stage 1, ≥90; stage 2, 60–89; stage 3, 30–59; stage 4, 15–29; stage 5, <15 (mL/min/1.73 m^2^) for group analyses, so they had not necessarily CKD state.

### 2.5. Statistical Analysis

All primary analyses were performed on an intention-to-treat basis. Data were expressed as the mean (±SD) or a percentage. As the distribution of the UACR was highly skewed, this parameter was log-transformed before the statistical analyses. The *χ* ^2^ test was used to evaluate differences in prevalence rates. Differences in BPs, eGFR, and UACR values at enrollment and at the end of the study were compared using a paired *t*-test. Differences in BPs, eGFR, and UACR between the control group and the added treatment group at the end of the study were compared using an unpaired *t*-test. Pearson's correlation coefficients were used to measure the relationships between continuous measures. Multiple linear regression analyses were performed to analyze the association between changes in the eGFR and UACR, adjusting for significant covariates [30] including body mass index (BMI), current smoking, drinking habits, diabetes mellitus, hyperlipidemia, change in morning SBP (SBP at the 6th month minus the baseline value), eGFR at baseline, and the use of renin angiotensin system inhibitors. Age and gender were not included because the MDRD equation includes these two parameters. The Kruskal Wallis test for the change in eGFR and UACR, and one-way analysis of variance for the change in morning SBP were performed to detect differences among different CKD stages. Associations/differences with a *P* value less than 0.05 (two-tailed) were considered to be statistically significant. All statistical analyses were performed with SPSS version 11 software (SPSS Inc., Chicago, IL).

## 3. Results

The randomization was successfully conducted and the baseline characteristics (except for hyperlipidemia), BPs, s-Cr, and eGFR, were similar between the control and the added treatment groups (Table 1). The mean dose of doxazosin at the end of the study was 3.4 ± 1.1 mg/day. A *β*-blocker (atenolol 25 mg/day for 154 patients or bisoprolol 5 mg/day for 1 patient) was added to 155 patients (50.3%) of the added treatment group because their morning SBPs did not reach the target level of 135 mmHg or less with doxazosin alone during the first 3 months of treatment after randomization.

### 3.1. Changes in BP, eGFR, and UACR

The morning and evening SBP decreased significantly more in the added treatment group than in the control group (morning SBP: −13.7 versus −5.2 mmHg, *P* < 0.01; evening SBP: −6.7 versus −3.0 mmHg, *P* = 0.048) (Table 2). Detailed results of the BP changes are described in our main paper [25].

eGFR was significantly decreased in the added treatment group and the control group from the baseline to 6 months (Table 2). However, the extent of the change in eGFR (the value at 6th months minus the baseline value) was significantly greater in the added treatment group than in the control group (−3.83 versus −1.08 mL/min/1.73 m^2^, *P* = 0.001).

The UACR was also significantly reduced in the added treatment group, and the median differences in UACR were −3.4 in the added treatment group and 0.0 mg/gCr in the control group (*P* < 0.001) from the baseline to the end of the study (Table 2).

The change in eGFR and the change in UACR were significantly associated with the use of doxazosin, and these associations were independent of changes in morning SBP and several other covariates (Table 3). The results were almost identical even after eliminating the four outliers of the change in eGFR or UACR (change in eGFR: *β* = −0.124, *P* = 0.003 for use of doxazosin; *β* = 0.063, *P* = 0.047 for change in morning systolic BP; change in UACR: *β* = −0.223, *P* < 0.001, for use of doxazosin; *β* = 0.204, *P* < 0.001 for change in morning systolic BP).

### 3.2. Association of the Changes in eGFR and UACR

In univariate analyses, the changes in eGFR from the baseline to the end of the study were associated with the change in UACR in the added treatment group (*r* = 0.18, *P* = 0.002), but not in the control group (*r* = 0.04, *P* = 0.52). In multivariable analyses adjusting for hyperlipidemia, diabetes mellitus, smoking, drinking, and changes in morning SBP and eGFR at baseline, these relationships remained significant (Table 4). The results were almost identical even after eliminating the four outliers of the change in eGFR or UACR (*β* = 0.196, *P* = 0.002 in the added treatment group; *β* = −0.002, *P* = 0.967 in the control group). When the data of CKD stage 1 were excluded, the results were essentially the same (*r* = 0.16, *P* = 0.033 in the added treatment group; *r* = 0.04, *P* = 0.55 in the control group).

### 3.3. Change in eGFR by CKD Stages

Next, the change in eGFR was separately analyzed by CKD stages 1 to 4.  Table 5 shows the extent of changes in eGFR at each CKD stage. In the control group, there were no significant differences of the change in eGFR between the different stages (*P* = 0.13). In the added treatment group, eGFR was significantly more decreased in stage 1 than in stages 2 to 4 (*P* = 0.002).

### 3.4. Change in UACR and Morning SBP at Each CKD Stage

The change in UACR and morning SBP were also separately analyzed at each CKD stage. There were no significant differences in the changes in UACR and morning SBP between different stages in either the added treatment group (*P* = 0.22, *P* = 0.55) or control group (*P* = 0.36, *P* = 0.39), respectively (Table 5).

## 4. Discussion

In this study, eGFR and UACR were significantly reduced by adrenergic blockers in patients with morning hypertension. The change in eGFR was significantly and independently associated with the change in UACR in the added treatment group. Our findings may help to clarify the renal effects of antihypertensive medication, especially antiadrenergic medication. In addition, the clinical significance of this study is that UACR was improved even though the eGFR was slightly reduced by adding antihypertensive medication.

### 4.1. Antihypertensive Therapy and Change in eGFR

In the present study, eGFR was reduced by adrenergic blockers over the 6-month treatment period. Several studies have demonstrated that eGFR can be reduced with calcium channel blockers, diuretics [22, 31], and angiotensin-converting enzyme inhibitors or angiotensin receptor blockers [32]. A Japanese study showed that a bedtime dose of doxazosin controlled morning BP but reduced eGFR [33]. However, the lack of a control group in that study prevented a definitive conclusion about the effect of doxazosin on the reduction in eGFR [33]. In the present study, eGFR was significantly reduced in the added treatment group compared with the control group, and the change in eGFR was significantly and independently associated with the use of doxazosin and the change in morning SBP, respectively. These results indicate that the reduction of eGFR was attributable to the BP reduction and doxazosin per se. One possible explanation for the reduction in eGFR is that it was due to a reduction in intraglomerular pressure induced by the decrease in systemic BP. The blocking of the systemic and renal sympathetic nervous system may have reduced GFR by dilating the renal arteries, which may thereby have protected the kidney.

### 4.2. The Relationship between the Change in eGFR and Change in UACR

In the present study, an independent and positive relationship between the change in eGFR and the UACR was observed in the added treatment group, but not in the control group. According to several studies, the hemodynamic load is the main determinant of the reduction in UACR [34–36] and GFR [37] in hypertension. BP reduction by antihypertensive treatments could unload the pressure in the renal glomeruli, which would contribute to a reduction in eGFR and UACR. Therefore, the reduction in eGFR by 6-month administration of adrenergic blockers could be due to unloading of the renal glomeruli.

### 4.3. Change in eGFR, UACR, and Morning SBP at Each CKD Stage

When the change in eGFR was separately analyzed by CKD stage, the extent of the change in eGFR in the added treatment group was significantly greater in stage 1 than in the other stages. No such difference was seen in the control group. The mean reduction in eGFR was 7 (mL/min/1.73 m^2^) in stage 1 and less than 3 (mL/min/1.73 m^2^) in stages 2 to 4 in the added treatment group. However, these small changes were not clinically significant. To the best of our knowledge, there has been no report showing that the change in eGFR due to antihypertensive treatment differs according to CKD stage. When the changes in UACR were similarly analyzed, no differences were seen among stages. Therefore, these findings suggest that the reduction in eGFR along with the reduction in UACR by adrenergic blockers would not require special attention, at least in the short term. In addition, it is speculated that the effects of the change in morning SBP on eGFR were different by the state of eGFR at the baseline.

### 4.4. The Effect of Alpha Blocker Doxazosin on the Renal Function

The alpha blocker has shown the renoprotective beyond the blood pressure lowering effect [38]. Efferent glomerular arteriolar vasoconstriction in response to endogenous norepinephrine is mediated by *α*_1_ adrenergic receptor, and this mechanism is cause of exaggerated in UACR [39]. The doxazosin has the ability to preserve renal autoregulation of renal blood flow and the observation of unaltered intraglomerular pressure. These results indicated that doxazosin has major preglomerular dilatory effects and reduces postglomerular resistance.

### 4.5. Study Limitations

There were several limitations in the present study. First, we could not employ the inulin clearance method, the gold standard for the measurement of GFR. Second, the MDRD equation could have underestimated the GFR, especially above 60 mL/min/1.73 m^2^. However, it has been shown that an eGFR over 60 mL/min/1.73 m^2^ can predict mortality [40, 41] and cardiorenal events [41] similar to an eGFR below 60 mL/min/1.73 m^2^. In addition, the change in eGFR reflects the accurate renal functional change even in the patients with eGFR over 60 mL/min/1.73 m^2^ [42]. Therefore, the change in eGFR in the patients with eGFR over 60 mL/min/1.73 m^2^ achieved some positive results. Indeed, the change of eGFR in CKD stage 1 might be within the error range of the MDRD equation or the effect of regression to the mean. However, the relation between the change in eGFR and that in UACR remained even when the results for patients with CKD stage 1 were eliminated. Therefore, we stand by our conclusion that a reduction of eGFR induced by adrenergic blockers does not require special attention in the short term. Third, the correlation between the change in eGFR and the change in UACR was relatively weak because of the heterogeneity of the patients' backgrounds. Fourth, the long-term effect of doxazosin on eGFR could not be clarified in this study. However, it has been shown that different antihypertensive combination therapies (a calcium-channel blocker plus an angiotensin converting-enzyme inhibitor (ACE-I) versus a diuretic plus an ACE-I) are associated with different levels of reduction of eGFR over a 3-month period, and after 3 months eGFR shows a gradual and parallel decline in both drug regimens [43]. Therefore, some positive results are also observed at sixth months. Fifth, the associations between the use of doxazosin and the change in eGFR or UACR were relatively weak. These weak associations are attributable to large differences in response to adrenergic blockers among individuals.

This study is a subanalysis of the JMS-1 Study; therefore, several table data of baseline were overlapped in a previous paper.

## 5. Conclusion

In patients with morning hypertension, both eGFR and UACR were reduced by the additional use of adrenergic blockers. These reductions were significantly and independently associated with the use of doxazosin and the reduction in SBP. The reduction of eGFR was significantly associated with that in UACR. In addition, the reduction of eGFR by the adrenergic blockers was slight and exclusively seen in the CKD stage 1 patients. These results indicate that the reduction of eGFR induced by adrenergic blockers does not require special attention in the short term and could predict an improvement of urinary albumin excretion in patients with morning hypertension.

## Figures and Tables

**Table 1:** Baseline characteristics of patients.

	Added treatment group (*n* = 308)	Control group (*n* = 303)	*P* value
Age (years)	70.2 ± 9.2	70.1 ± 10.0	0.88
Male (%)	45.1	43.6	0.76
Body mass index (kg/m^2^)	24.1 ± 3.3	24.2 ± 3.6	0.82
Duration of hypertension (years)	11.5 ± 9.6	11.3 ± 8.7	0.74
Duration of hypertensive therapy (years)	8.6 ± 8.3	8.0 ± 7.1	0.34
Current smokers (%)	19.2	18.8	1.00
Habitual drinkers (%)	34.1	30.0	0.32
Diabetes or impaired glucose tolerance (%)	15.3	16.5	0.76
Hyperlipidemia (%)	32.5	40.9	0.04
Number of antihypertensive drugs	1.6 ± 0.8	1.6 ± 0.7	0.71
Calcium channel blockers (%)	66.6	65.6	0.82
Angiotensin-converting enzyme inhibitors (%)	13.1	15.6	0.37
Angiotensin II receptor blockers (%)	60.3	57.5	0.54
Diuretics (%)	22.0	21.9	0.93
Oral drugs for diabetes mellitus (%)	9.5	7.6	0.51
Medication for hyperlipidemia (%)	15.1	13.6	0.62
Office SBP (mmHg)	157 ± 18	156 ± 19	0.43
Office DBP (mmHg)	83 ± 11	82 ± 12	0.92
Office pulse rate (bpm)	73 ± 18	72 ± 17	0.11

Data are shown as the means ± SD. Urinary albumin [values are] expressed as the median [] (25% value, 75% value). BP: blood pressure; bpm: beats per minute; SBP: systolic blood pressure; DBP: diastolic blood pressure.

**Table 2:** Blood pressure and renal functional parameter changes during 6 months.

	Added treatment group (*n* = 308)		Control group (*n* = 303)	
	baseline	6th month	baseline	6th month
Morning SBP (mmHg)	153 ± 13	140 ± 15^∗∗,††^	151 ± 15	146 ± 16**
Morning DBP (mmHg)	82 ± 10	73 ± 10^∗∗,††^	82 ± 11	80 ± 11**
Evening SBP (mmHg)	140 ± 14	133 ± 15^∗∗,††^	140 ± 17	137 ± 15**
Evening DBP (mmHg)	75 ± 10	69 ± 10^∗∗,††^	76 ± 11	74 ± 10**
Estimated GFR (mL/min/1.73 m^2^)	84.1 ± 21.1	80.3 ± 19.3**	82.0 ± 18.9	80.9 ± 19.9*
Creatinine (mg/dL)	0.79 ± 0.27	0.82 ± 0.29**	0.80 ± 0.25	0.82 ± 0.28*
UACR (mg/gCr)	23.0 (11.4–63.1)	14.7 (8.2–35.8)^∗∗,†^	21.0 (10.7–53.2)	19.4 (11.5–53.6)

Data are shown as the means ± SD. UACR is expressed as the median values (25% value–75% value). BP: blood pressure; SBP: systolic blood pressure; DBP: diastolic blood pressure; GFR: glomerular filtration rate; UACR: urinary albumin/creatinine ratio.

*P < 0.05, **P < 0.001 versus baseline in each group: ^†^ *P* < 0.05, ^††^ *P* < 0.001 versus the 6th month in the control group.

**Table 3:** Factors associated with change in eGFR and UACR.

Variable	Change in eGFR (6th month minus baseline)		Change in UACR (6th month minus baseline)	
	**β** coefficient	*P* value	**β** coefficient	*P* value
Use of doxazosin (use = 1, no use = 0)	−0.107	0.012	−0.178	<0.001
Change in morning systolic BP (6th month minus baseline)	0.085	0.045	0.251	<0.001
Adjusted *R* ^2^	0.101		0.117	

Adjustments were made for body mass index, hyperlipidemia (Yes = 1; No = 0), diabetes mellitus (Yes = 1; No = 0), smoking habit (Yes = 1; No = 0), drinking habit (Yes = 1; No = 0), and change in morning systolic BP.

eGFR: estimated glomerular filtration rate. UACR: urinary albumin to creatinine ratio. BP: blood pressure. *R* ^2^: determination coefficient.

**Table 4:** Factors associated with change in eGFR.

Variable	Change in UACR (6th month minus baseline)			
	Added treatment group (*n* = 308)		Control group (*n* = 303)	
	**β** coefficient	*P* value	**β** coefficient	*P* value
Change in eGFR (6th month minus baseline)	0.198	0.001	−0.002	0.966
Adjusted *R* ^2^	0.093		0.115	

Adjustments were made for body mass index, hyperlipidemia (Yes = 1; No = 0), diabetes mellitus (Yes = 1; No = 0), smoking habit (Yes = 1; No = 0), drinking habit (Yes = 1; No = 0), change in morning systolic BP, eGFR at baseline, and the use of renin angiotensin system inhibitors.

UACR: urinary albumin to creatinine ratio. eGFR: estimated glomerular filtration rate. BP: blood pressure. *R* ^2^: determination coefficient.

**Table 5:** Change in eGFR, UACR and morning SBP at each CKD stage.

CKD stage	Change in eGFR (6th month minus baseline)		Change in UACR (6th month minus baseline)		Change in morning SBP (6th month minus baseline)	
	Added treatment group (*n* = 308)	Control group (*n* = 303)	Added treatment group (*n* = 308)	Control group (*n* = 303)	Added treatment group (*n* = 308)	Control group (*n* = 303)
Stage 1	−6.4 ± 15.5	−2.0 ± 9.8	−4.3 (−16.9, 0.0)	−0.05 (−6.7, 4.4)	−14 ± 14	−5.3 ± 12
Stage 2	−2.5 ± 7.5	−0.4 ± 7.9	−3.2 (−20.8, 0.0)	0 (−7.3, 8.5)	−12 ± 14	−3.2 ± 15
Stage 3 + stage 4	−2.0 ± 4.3	−1.5 ± 5.8	0 (−27.0, 1.1)	0 (−19.2, 13.9)	−12 ± 12	−5.5 ± 13
*P* value	0.002	0.13	0.22	0.36	0.55	0.39

Changes in eGFR are shown as the means ± SD. Changes in UACR are expressed as the median values (25% value, 75% value). eGFR: estimated glomerular filtration rate; UACR: urinary albumin/creatinine ratio; CKD: chronic kidney disease; SBP: systolic blood pressure.

The numbers of patients per CKD stage are 111, 166, and 31 for CKD stages 1, 2, and 3 + 4 in the added treatment group and 104, 172, and 26 for these stages in the control group, respectively. The *P* values were calculated by the Kruskal Wallis test for the change in eGFR and UACR and one-way analysis of variance for the change in morning SBP.

## References

[1] Collins AJ, Couser WG, Dirks JH (2006). World Kidney Day: an idea whose time has come. *Kidney International*.

[2] National Kidney Foundation. K/DOQI clinical practice guidelines for chronic kidney disease (2002). Evaluation, classification, and stratification. *American Journal of Kidney Diseases*.

[3] Sarnak MJ, Levey AS, Schoolwerth AC (2003). Kidney disease as a risk factor for development of cardiovascular disease: a statement from the American Heart Association Councils on Kidney in Cardiovascular Disease, High Blood Pressure Research, Clinical Cardiology, and Epidemiology and Prevention. *Circulation*.

[4] Ninomiya T, Kiyohara Y, Kubo M (2005). Chronic kidney disease and cardiovascular disease in a general Japanese population: the Hisayama Study. *Kidney International*.

[5] Nakayama M, Metoki H, Terawaki H (2007). Kidney dysfunction as a risk factor for first symptomatic stroke events in a general Japanese population—the Ohasama study. *Nephrology Dialysis Transplantation*.

[6] Anavekar NS, McMurray JJV, Velazquez EJ (2004). Relation between renal dysfunction and cardiovascular outcomes after myocardial infarction. *The New England Journal of Medicine*.

[7] Kottgen A, Russell SD, Loehr LR (2007). Reduced kidney function as a risk factor for incident heart failure: the Atherosclerosis Risk in Communities (ARIC) Study. *Journal of the American Society of Nephrology*.

[8] Go AS, Chertow GM, Fan D, McCulloch CE, Hsu C-Y (2004). Chronic kidney disease and the risks of death, cardiovascular events, and hospitalization. *The New England Journal of Medicine*.

[9] Irie F, Iso H, Sairenchi T (2006). The relationships of proteinuria, serum creatinine, glomerular filtration rate with cardiovascular disease mortality in Japanese general population. *Kidney International*.

[10] Klag MJ, Whelton PK, Randall BL (1996). Blood pressure and end-stage renal disease in men. *The New England Journal of Medicine*.

[11] Klag MJ, Whelton PK, Randall BL, Neaton JD, Brancati FL, Stamler J (1997). End-stage renal disease in African-American and white men: 16-year MRFIT findings. *Journal of the American Medical Association*.

[12] Tozawa M, Iseki K, Iseki C, Kinjo K, Ikemiya Y, Takishita S (2003). Blood pressure predicts risk of developing end-stage renal disease in men and women. *Hypertension*.

[13] Yamagata K, Ishida K, Sairenchi T (2007). Risk factors for chronic kidney disease in a community-based population: a 10-year follow-up study. *Kidney International*.

[14] Ishikawa J, Hoshide S, Shibasaki S (2008). Relationship between morning hypertension identified by home blood pressure monitoring and brain natriuretic peptide and estimated glomerular filtration rate: the Japan morning surge 1 (JMS-1) study. *Journal of Clinical Hypertension*.

[15] Suzuki H, Nakamoto H, Okada H, Sugahara S, Kanno Y (2002). Self-measured systolic blood pressure in the morning is a strong indicator of decline of renal function in hypertensive patients with non-diabetic chronic renal insufficiency. *Clinical and Experimental Hypertension*.

[16] Marfella R, Gualdiero P, Siniscalchi M (2003). Morning blood pressure peak, QT intervals, and sympathetic activity in hypertensive patients. *Hypertension*.

[17] Kario K, Pickering TG, Hoshide S (2004). Morning blood pressure surge and hypertensive cerebrovascular disease: role of the alpha adrenergic sympathetic nervous system. *American Journal of Hypertension*.

[18] Esler MD, Krum H, Sobotka PA (2010). Renal sympathetic denervation in patients with treatment-resistant hypertension (The Symplicity HTN-2 Trial): a randomised controlled trial. *The Lancet*.

[19] Ogihara T, Kikuchi K, Matsuoka H (2009). The japanese society of hypertension guidelines for the management of hypertension (JSH 2009). *Hypertension Research*.

[20] Andersen S, Tarnow L, Rossing P, Hansen BV, Parving H-H (2000). Renoprotective effects of angiotensin II receptor blockade in type 1 diabetic patients with diabetic nephropathy. *Kidney International*.

[21] Svarstad E, Gerdts E, Omvik P, Ofstad J, Iversen BM (2001). Renal hemodynamic effects of captopril and doxazosin during slight physical activity in hypertensive patients with type-1 diabetes mellitus. *Kidney and Blood Pressure Research*.

[22] Bakris GL, Toto RD, McCullough PA, Rocha R, Purkayastha D, Davis P (2008). Effects of different ACE inhibitor combinations on albuminuria: results of the GUARD study. *Kidney International*.

[23] O’Donnell MJ, Rowe BR, Lawson N, Horton A, Gyde OH, Barnett AH (1993). Comparison of the effects of an angiotensin converting enzyme inhibitor and a calcium antagonist in hypertensive, macroproteinuric diabetic patients: a randomised double-blind study. *Journal of Human Hypertension*.

[24] Ishikawa J, Hoshide S, Shibasaki S (2006). The japan morning surge-1 (JMS-1) study: protocol description. *Hypertension Research*.

[25] Kario K, Matsui Y, Shibasaki S (2008). An *α*-adrenergic blocker titrated by self-measured blood pressure recordings lowered blood pressure and microalbuminuria in patients with morning hypertension: the japan morning surge-1 study. *Journal of Hypertension*.

[26] Anwar YA, Giacco S, McCabe EJ, Tendler BE, White WB (1998). Evaluation of the efficacy of the Omron HEM-737 intellisense device for use on adults according to the recommendations of the Association for the Advancement of Medical Instrumentation. *Blood Pressure Monitoring*.

[27] Imai Y, Otsuka K, Kawano Y (2003). Japanese Society of Hypertension (JSH) guidelines for self-monitoring of blood pressure at home. *Hypertension Research*.

[28] Imai E, Horio M, Nitta K (2007). Estimation of glomerular filtration rate by the MDRD study equation modified for Japanese patients with chronic kidney disease. *Clinical and Experimental Nephrology*.

[29] Levey AS, Eckardt K-U, Tsukamoto Y (2005). Definition and classification of chronic kidney disease: a position statement from Kidney Disease: Improving Global Outcomes (KDIGO). *Kidney International*.

[30] Rosa TT, Palatini P (2000). Clinical value of microalbuminuria in hypertension. *Journal of Hypertension*.

[31] Schrier RW, Estacio RO, Esler A, Mehler P (2002). Effects of aggressive blood pressure control in normotensive type 2 diabetic patients on albuminuria, retinopathy and strokes. *Kidney International*.

[32] Rossing K, Schjoedt KJ, Jensen BR, Boomsma F, Parving H-H (2005). Enhanced renoprotective effects of ultrahigh doses of irbesartan in patients with type 2 diabetes and microalbuminuria. *Kidney International*.

[33] Ikeda T, Gomi T, Shibuya Y, Shinozaki S, Suzuki Y, Matsuda N (2007). Add-on effect of bedtime dosing of the *α*1-adrenergic receptor antagonist doxazosin on morning hypertension and left ventricular hypertrophy in patients undergoing long-term amlodipine monotherapy. *Hypertension Research*.

[34] Pedrinelli R (1996). Microalbuminuria in Hypertension. *Nephron*.

[35] Palatini P, Mormino P, Mos L (2005). Microalbuminuria, renal function and development of sustained hypertension: a longitudinal study in the early stage of hypertension. *Journal of Hypertension*.

[36] Pedrinelli R, Penno G, Dell’Omo G (1999). Microalbuminuria and transcapillary albumin leakage in essential hypertension. *Hypertension*.

[37] Apperloo AJ, De Zeeuw D, De Jong PE (1997). A short-term antihypertensive treatment-induced fall in glomerular filtration rate predicts long-term stability of renal function. *Kidney International*.

[38] Jyothirmayi GN, Alluru I, Reddi AS (1996). Doxazosin prevents proteinuria and glomerular loss of heparan sulfate in diabetic rats. *Hypertension*.

[39] Van Brummelen P, Jie K, Van Zwieten PA (1986). *α*-Adrenergic receptors in human blood vessels. *British Journal of Clinical Pharmacology*.

[40] James MT, Hemmelgarn BR, Wiebe N (2010). Glomerular filtration rate, proteinuria, and the incidence and consequences of acute kidney injury: a cohort study. *The Lancet*.

[41] Viazzi F, Leoncini G, Conti N (2010). Combined effect of albuminuria and estimated glomerular filtration rate on cardiovascular events and all-cause mortality in uncomplicated hypertensive patients. *Journal of Hypertension*.

[42] Ruggenenti P, Perticucci E, Cravedi P (2008). Role of remission clinics in the longitudinal treatment of CKD. *Journal of the American Society of Nephrology*.

[43] Heerspink HL, de Zeeuw D (2010). Composite renal endpoints: was ACCOMPLISH accomplished?. *The Lancet*.

